# Longitudinal study on *Plasmodium falciparum* gametocyte carriage following artemether-lumefantrine administration in a cohort of children aged 12–47 months living in Western Kenya, a high transmission area

**DOI:** 10.1186/1475-2875-13-265

**Published:** 2014-07-09

**Authors:** Ben Andagalu, Joan Mativo, Edwin Kamau, Bernhards Ogutu

**Affiliations:** 1 Department of Emerging Infectious Diseases-Global Emerging Infections Surveillance and Response System (DEID-GEIS) Program, United States Army Medical Research Unit-Kenya (USAMRU-K), Kenya Medical Research Institute (KEMRI)-Walter Reed Project, P.O. Box 54, Kisumu, 40100, Kenya

**Keywords:** Gametocytes, Western Kenya, Longitudinal study, Asymptomatic children, Artemether-lumefantrine, Epidemiological study

## Abstract

**Background:**

The effects that artemether-lumefantrine (AL) has on gametocyte dynamics in the short-term have recently been described. However there is limited long-term longitudinal data on the effect of AL on gametocyte dynamics in asymptomatic children.

**Methods:**

An epidemiological study was conducted in Kombewa, Western Kenya, in which 270 asymptomatic children aged between 12 and 47 months were enrolled. The subjects were randomized to receive either a course of AL or placebo at enrolment. Active follow-up was conducted for one year.

**Results:**

The gametocyte prevalence and density dynamics throughout the study period mirrored that of the asexual forms. The proportion of initially parasitaemic subjects becoming gametocytaemic was significantly lower in the AL arm for the first 12 weeks following randomization. The geometric mean gametocyte density was lower in the AL arm for 2 weeks following randomization. None of the variables of interest had a statistically significant effect on the duration of gametocytaemia. There is no effect seen in subjects who are not parasitaemic at the time of drug administration.

**Conclusions:**

The treatment of asymptomatic parasitaemic subjects with AL results in a significant reduction in the proportion of subjects who become gametocytaemic for at least 12 weeks.

## Background

Both symptomatic and asymptomatic malaria infected persons tend to harbour gametocytes, the sexual stage of the malaria parasite that mediates its transmission from the infected human host to a susceptible mosquito [1]. Because they are less likely to seek treatment, the asymptomatic carriers constitute a significant reservoir of parasites available for transmission by mosquitoes [2]. The proportion of asymptomatic or subclinical *Plasmodium falciparum* infections in endemic countries is high, with microscopically detected prevalence reported to be as high as 39% [3,4]. A large number of gametocyte carriers have submicroscopic gametocytaemia [4,5]. Studies have shown that molecular methods detect gametocytes 3- to 10-fold more than microscopy [6-8]. Interestingly, even at submicroscopic densities, gametocytes do infect mosquitoes and contribute to malaria transmission [9,10].

Gametocyte carriage in naturally infected individuals in a population is influenced by many factors such as the age group [5,11-13], transmission settings [5,14,15], seasons [5,16-18] and the human host genetic factors [19-21]. Other important risk factors for gametocyte carriage include anaemia [22], durations of the infections, recrudescent infections [23], fever [24], mixed infections [23,25] and drug treatment [26,27].

Data on duration of gametocytes carriage in natural infections is limited because such longitudinal studies are challenging to conduct [5]. In a longitudinal study conducted in 1958, volunteers were followed up for one year, with sampling of the parasites done every other day [28]. Eighty percent of the enrolled subjects had detectable gametocytes by microscopy at least once during follow up, with more than half carrying gametocytes 20 times or more, and the longest duration of carriage was estimated at 188 days. In another study, gametocyte carriage was shown to extend to 30 days in Gambian adults in a single transmission season [11]. In a study conducted in western Kenya on asymptomatic children, the mean duration of gametocyte carriage was shown to decrease with increasing age [27]. This was a 30-day longitudinal study. Interestingly, children of < 10 years could carry gametocytes for three weeks or more.

Treatment of asymptomatic asexual *P. falciparum* carriers directly benefits the carriers given that the clearance of the asexual parasite load prevents anaemia, which in turn is associated with reduced work capacity, poor pregnancy outcomes and reduced cognitive function [2]. Adoption of artemisinin‒based combination therapy (ACT) as the first-line treatment of uncomplicated malaria has been beneficial because of their effect on gametocytes and consequently malaria transmission [29-32]. Artemether-lumefantrine (AL) is one of the most widely used ACT in Africa with extensive efficacy and safety data [2]. The effects that AL has on gametocyte dynamics in the short-term have recently been described [2,33]. However, there is limited long-term longitudinal data on the effect that the administration of AL to asymptomatic *P. falciparum* carriers has on gametocyte dynamics. To describe this, data collected from a longitudinal cohort study of the epidemiology of paediatric malaria in Kombewa Division, Western Kenya was analysed.

## Methods

### Source of data

Data was obtained from the longitudinal cohort study of the epidemiology of paediatric malaria in Kombewa Division, Western Kenya. The study was conducted in order to estimate the prevalence of asymptomatic and symptomatic malaria as well as to describe the temporal pattern of episodes of symptomatic malaria among young children living in Kombewa Division. An additional aim was to assess whether administration of a curative dose of an anti-malarial treatment at the beginning of the study leads to a large change in the pattern of development of episodes of malaria during the subsequent follow-up period. The information obtained from this study was for the planning and design of a paediatric malaria vaccine trial that was to be conducted in the same area.

### Study area

Kombewa Division is a 361 square kilometre rural area located near Lake Victoria in the western part of Kenya, with a population of approximately 70,000 and an under-five population of approximately 8,500. The climate in Kombewa is characterized by two rainy seasons – the long rains (April through June) and the short rains (August through October). The vegetation is largely savanna in nature. Kombewa has year round transmission of malaria, with the most intense transmission occurring during the long rains and the short rains. For the purpose of organizing recruitment and follow-up of the volunteers, the field site was divided into 22 subdivisions. Within each subdivision in which a participant resided there was a satellite field site that was staffed 24 hrs/day, 7 days/week. Each household participating in the study was thus located within a 1-mile radius of a satellite field site that was accessible during the rainy and dry seasons. The study protocol was reviewed and approved by the Ethical Review Committee of the Kenya Medical Research Institute (KEMRI) and the Human Use Review Committee of the Walter Reed Army Institute of Research (WRAIR) and by the Human Subjects Research and Review Board of the Surgeon General of the US Army at Fort Detrick, Maryland. Parents or guardians of participants were provided written, informed consent before screening and enrolment and had to pass an assessment of understanding.

### Study design and procedures

This was an epidemiological follow-up study, in which a total of 270 initially healthy, asymptomatic children aged between 12 months and 47 months living within the study area were enrolled in three age cohorts of 12–23 months, 24–35 months and 36–47 months. Inclusion criteria included being 12 to 48 months of age, verifiable by appropriate documentation. Subjects with clinical signs of malaria and other significant medical conditions such as homozygous sickle cell disease and malnutrition were excluded. Samples for a baseline malaria blood film (MBF) and a full blood count were collected just before randomization; the results of MBF were not used to determine eligibility, and were not made available to the investigators. The children were then randomized to receive either a course of artemether-lumefantrine (Coartem®) or placebo using a list prepared in advance using a blocked randomization scheme. All study staff with the exception of the study pharmacists and the study coordinator were blinded to the allocations. The treatment was prepared by the study pharmacist at the clinic and its administration directly supervised by study team members – the first, third and fifth doses were administered at the clinic, while the second, fourth and sixth doses were administered by field workers at home. Each of the children was then followed up for one year. The follow-up consisted of active surveillance and passive surveillance. Active surveillance consisted of weekly visits by field workers to the participants’ homes and scheduled monthly visits by the participants to the study clinic, during which samples for malaria blood films were collected. Haemoglobin determination was done during the monthly visit at the clinic. The results of the MBFs collected during the active surveillance were not availed to the investigators unless the subject was unwell during a scheduled contact. Passive surveillance consisted of unscheduled sick visits by the study participants to the study clinic whenever the participants had specific complaints – during these visits, laboratory assessments including malaria blood films were done whenever the clinical condition warranted. The results of the MBFs taken during sick visits were made available to the investigators. Subjects who were symptomatic and were diagnosed with malaria as per the laboratory assessment during the follow up period were treated with AL, regardless of the randomization group.

### Diagnosis of malaria

Duplicate thick and thin blood smears were made and air dried. Thin blood smear was methanol fixed and both smears were stained with 5% Giemsa in phosphate buffer for 30 minutes. With high power objective the smears were examined, the number, species and stage of the parasites were recorded. The total numbers of parasites, both asexual and sexual forms per high power field (HPF) were counted against 200 white blood cells. For samples that had high density parasitaemia, the asexual forms were counted against 2,000 red blood cells. A total of 100 HPF were examined before a slide was considered negative, and a minimum of 25HPF were examined on a positive smear. Two independent microscopists examined the MBFs, and a mean of their findings calculated. Discrepant results were resolved by having a third reader, and the two closest results were taken. The microscopists were proficient and worked under the guidance of malaria diagnostic centre within the programme.

### Statistical analysis

All data were entered and verified in Microsoft Access then imported into STATA software version 10 (StataCorp., College Station, TX). Malaria parasite densities were converted from parasites per 200 white blood cells and parasites per 2,000 red blood cells to parasite/μL, assuming a WBC count of 8000 or a RBC count of 4×10^6^ respectively. The duration of gametocytaemia was calculated by assuming that a single gametocyte positive slide represented gametocytes circulating in the peripheral blood for at least 2.5 days. If participants presented with gametocyte positive MBFs on successive visits, the duration of gametocytaemia was estimated as the interval between the visits plus 2.5 days [34,35]. Covariates of interest were the use of mosquito nets, age, presence of mixed infection (*P. falciparum* and *Plasmodium malariae*, or *P. falciparum* and *Plasmodium ovale*) and whether or not the child was pre-treated with AL at randomization. The baseline characteristics data is presented of the three age groups; 12–23, 24–35 and 36–47 months in that order respectively. The association between the time to the first or only episode of gametocytaemia, as well as the risk for multiple events of gametocytaemia, and the variables of interest was analysed using Cox regression analysis. The association between continuous outcomes (gametocyte density, gametocytaemia duration) and the outcomes of interest were evaluated using robust regression models.

## Results

### Study profile

A total of 539 children were screened, 270 met the eligibility criteria and were enrolled into the study where 135 were randomized to receive placebo and 135 randomized to receive AL. Of the 270 volunteers that were enrolled into the study, 69% (187) completed the study which the follow-up went on for a year. Of those that completed the study, 53% (99 of 187) were in the placebo arm and 47% (88 of 187) in the AL arm. Of the 83 volunteers that did not complete the one year follow-up, 81% (67) withdrew their consent, with 55% (46 of 83) participants citing their discomfort with the weekly blood draws as the reason for consent withdrawal. Migration out of the study area accounted for the remainder of those who did not complete the study. No deaths were recorded among the study cohort for the duration of the study. However, two serious adverse events (SAEs) were recorded where one child in the AL arm was hospitalized with a diagnosis of severe malaria 19 weeks after randomization - this particular participant had defaulted on clinic visits, while the other child in the placebo arm was hospitalized with severe pneumonia. Both children recovered. All children completed the six doses of the study treatment.

### Baseline characteristics

The baseline characteristics of both treatment arms were generally well balanced (Table 1). Insecticide-treated nets (ITN) use was low in the study cohort (33% overall) with a slight predominance in the placebo arm (36.3% versus. 29.6%). There was a general trend of higher ITN use in the 12–23 months age group than the older age groups (overall 43.3% versus 30.0% and 25.6%). Parasitological data for the randomization day was available for 265 subjects. The prevalence of parasitaemia at randomization was expectedly high, considering that enrolment took place over a two-week period between the end of May and early June, coinciding with the peak of the malaria season (Table 2). A total of 66.4% (176 of 265) participants had asexual parasitaemia at randomization. The prevalence was lower in bed-net users (58.1% versus 70.4%) and in the youngest age group (52.0% versus 72.0% and 76.1%). Gametocyte prevalence at enrolment was 17.4%, and was higher in bed net users (23.3% versus 15.0%) and lower in the youngest age group (16.0% versus 19.3% and 17.1%). The geometric mean asexual parasite density at enrolment was 3328 p/μL (IQR 940 – 12260) while the geometric mean gametocyte density was 94.1 p/μL (IQR 40 – 120), with little variation between the various groups.

**Table 1:** Baseline characteristics

	**AL (N = 135)**	**Placebo (N = 135)**	**Overall (270)**
**Age in months – Mean (SD)**^**a**^	29.6 (10.2)	30.1 (10.1)	29.8 (10.1)
**Male – N (%)**	72/135 (53.3)	69/135 (51.1)	141/270 (52.2)
**Hb - Mean (SD)**	9.9 (1.3)	9.9 (1.4)	9.9 (1.4)
**Bed net use overall – N (%)**	40/135 (29.6)	49/135 (36.3)	89/270 (33)
**Bed net use 12–23 months – N (%)**	16/45 (35.6)	23/45 (51.1)	39/45 (43.3)
**Bed net use 24–35 months – N (%)**	15/45 (33.3)	12/45 (26.7)	27/45 (30.0)
**Bed net use 36–47 months – N (%)**	9/45 (20.0)	14/45 (31.1)	23/45 (25.6)

^a^The standard deviation (SD) from the mean of the sample size analysed.

**Table 2:** Parasitological data at enrolment

	**N**	**N (%)**^**a**^	**p/μL, (IQR)**^**b**^	**N (%)**^**c**^	**p/μL, (IQR)**^**d**^
Overall	265	176/265 (66.4)	3328 (940,12260)	46/265 (17.4)	94.1 (40,120)
AL	133	92/133 (69.2)	4016.0 (1180,1500)	31/133 (23.3)	102.0 (40,120)
Placebo	132	84/132 (64.0)	2710.3 (860,10660)	15/132 (11.4)	80.0 (40,120)
Bed net users	86	50/86 (58.1)	2916.0 (880,10440)	20/86 (23.3)	112.0 (40,140)
Bed net non users	179	126/179 (70.4)	3509.0 (960,13080)	26/179 (15.0)	83.0 (40,120)
Age 12–23 mo	89	46/89 (52.0)	3296.0 (840,14560)	14/89 (16)	100.0 (40,120)
Age 24–35 mo	88	63/88 (72.0)	3067.0 (720,10480)	17/88 (19.3)	101.0 (40,120)
Age 36–47 mo	88	67/88 (76.1)	3620.3 (1440,12480)	15/88 (17.1)	75.2 (40,120)
Anemic	60	42/60 (70)	4373.3 (1760,13360)	8/60 (13.3)	115.4 (40,100)
Not anemic	194	128/194 (66.0)	3117.0 (780,12720)	37/194 (19.1)	92.2 (40,120)

^a^Asexual parasite prevalence.

^b^Asexual parasite density (p/μL), Geometric mean (Inter Quartile Range [IQR]).

^c^Gametocyte prevalence N (%).

^d^Gametocyte density (p/μL), Geometric mean (Inter Quartile Range [IQR]).

### Gametocyte prevalence and risk factors

**Figure 1:**
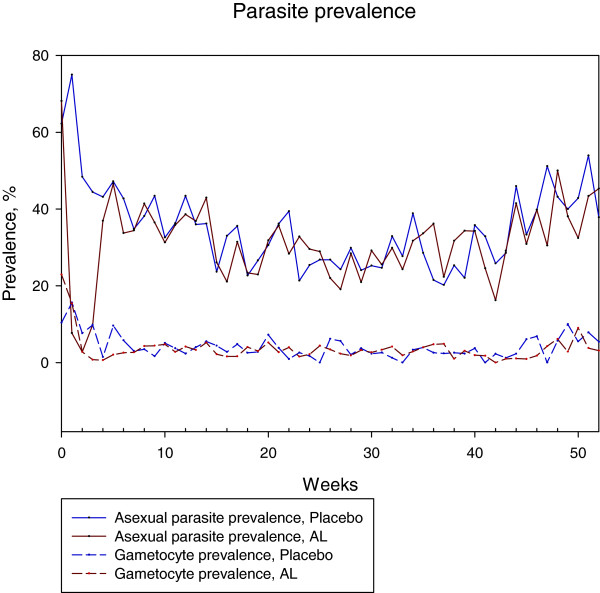
**Gametocyte prevalence dynamics.** Data showing the percent prevalence of asexual and sexual parasites throughout the study period. The highest prevalence of asexual and sexual parasites was 66.4% and 17.4% respectively which was at the beginning of the study. This coincided with height of malaria transmission season. The prevalence of sexual parasite mirrored asexual parasite throughout the study period.

**Figure 2:**
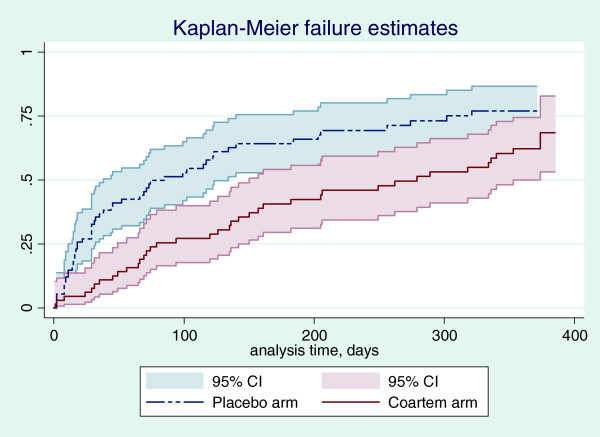
**Kaplan-Meier failure estimates showing the proportion of initially parasitaemic participants becoming gametocytaemic.** Participant who received AL at randomization had significantly lower gametocytes compared to placebo for the first 12 weeks.

The gametocyte prevalence dynamics throughout the study period were similar to the asexual parasite prevalence dynamics, with the gametocyte prevalence peaks coinciding with the asexual parasite prevalence peaks (Figure 1). For subjects who had a positive MBF at enrolment, the proportion of subjects who became gametocytaemic was significantly lower for those who received AL at randomization (24.6% versus. 50.7%, *P* = 0.002). This effect was observed for the first 12 weeks following randomization (Figure 2). For subjects who had a negative malaria smear at enrolment, the difference in the proportion of subjects who became gametocytaemic over the first 12 weeks in the placebo and AL arms was not statistically significant (11.6% versus 5.4% respectively, *P* = 0.326). Being in the older age category, AL administration at randomization and bed net use were protective against gametocytaemia, while being in the second age category, having a positive smear at enrolment and hyper-parasitaemia were significantly associated with gametocytaemia (Table 3, *P* < 0.001 and *P* = 0.034). A positive malaria smear at enrolment and mixed infection were associated with faster appearance of the first or only episode of gametocytaemia (Table 3, *P* = 0.016 and *P* = 0.001). A positive smear for malaria at enrolment and anaemia was statistically significantly associated with the development of multiple events of gametocytaemia (Table 3, both events *P* < 0.001), while the second age category, hyper-parasitaemia and mixed infection seemed to reduce the risk for multiple events (Table 3, *P* = 0.01 and *P* = 0.007).

**Table 3:** Gametocyte risk factors

**Variables**	**Gametocytaemia versus variables**	**First event versus variables**	**Multiple events versus variables**
	**OR (95% CI) p-value**	**HR (95% CI) p-value**	**HR (95% CI) p-value**
Age category 2	1.05 (1.00, 1.11) 0.036	0.44 (0.16, 1.23) 0.118	0.63 (0.44, 0.89) 0.01
Age category 3	0.36 (0.17, 0.74) 0.005	0.75 (0.29, 1.90) 0.54	0.76 (0.54, 0.89) 0.01
Bed net use	0.26 (0.08, 0.91) 0.035	1.32 (0.57, 3.05) 0.515	1.34 (1.01, 1.80) 0.46
Pf enrolment^a^	4.46 (2.94, 6.74) <0.001	3.36 (1.26, 8.98) 0.016	3.56 (2.37, 5.37) <0.001
Anaemia	1.24 (0.82, 1.88) 0.299	1.46 (0.55, 3.87) 0.447	2.06 (1.49, 2.85) <0.001
Hyper Pf^b^	1.44 (1.03, 2.01) 0.034	0.86 (0.20, 3.78) 0.845	0.34 (0.16, 0.74) 0.007
Pretreatment	1.81 (1.20, 2.72) 0.004	0.85 (0.38, 1.88) 0.682	0.81 (0.62, 1.06) 0.128
Mixed Infection	1.05 (1.00, 1.11) 0.036	4.89 (1.99, 11.99) 0.001	0.63 (0.44, 0.89) 0.01

^*a*^*Plasmodium falciparum* positive participants at enrolment.

^b^Participants with hyper parasitaemia which is 100,000 p/mL and above.

Odds ratio (OR), hazardous ratio (HR), the 95% confidence interval (95% CI) and p-values based on Cox regression analysis were generated. Association between gametocytaemia, the time to the first or only episode of gametocytaemia, the risk for multiple events of gametocytaemia, with the variables of interest were determined.

### Gametocyte density and duration

**Figure 3:**
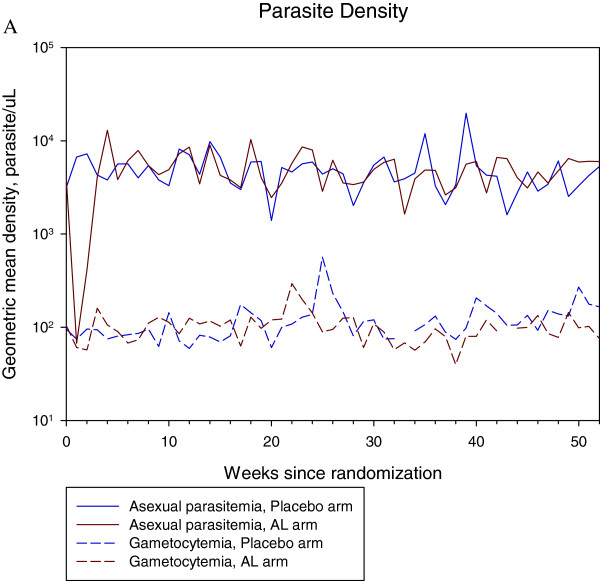
The dynamics of sexual and asexual parasite density: shows the dynamic of asexual and sexual parasite dynamics of both AL and placebo arms throughout the study.

**Figure 4:**
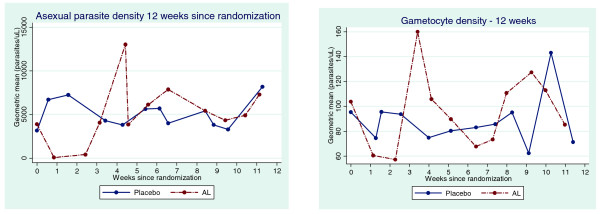
The dynamics of sexual and asexual parasite density: shows the dynamics of asexual and sexual parasite dynamics of both AL and placebo arms in the first 12 weeks of randomization.

The gametocyte density dynamics were similar to the asexual parasite density dynamics, with the peaks coinciding with the asexual parasite density peaks (Figure 3). The geometric mean density of asexual as well as sexual forms of the parasite was lower in the AL arm for 2 weeks following randomization. However, the densities actually surpassed those in the placebo arm after the third week before they returned to levels similar to those in the placebo arm (Figure 4). Gametocyte density was positively associated with asexual parasite density (Table 4, *P* = 0.008) and negatively associated with haemoglobin levels (Table 4, *P* < 0.001). The mean gametocytaemia duration was 7.4 days (range 2.5 – 41.5) in the lowest age category, 5.6 days (range 2.5 – 37.5) in the middle age category and 6.2 days (range 2.5 – 44.5) in the oldest age category. There was no statistically significant difference in the gametocytaemia duration between the age categories groups as determined by one-way ANOVA (F(2,443) = 2.11, *P* = 0.12).

**Table 4:** Gametocyte density analysis

**Variables**	**Gametocyte density versus variables**	***P *** **value**
	**Coef (95% CI)**	
Age	-0.327 (-0.840, 0.185)	0.211
Parasitaemia	0.0000364 (9.33E-06, 6.36E-05)	0.008
Hb	-8.009 (-12.355, -3.66334)	<0.001
Mixed Infection	-4.167 (-21.569, 13.236)	0.639
Age	-0.388 (-0.896, 0.121)	0.135
Parasitaemia	3.29E-05 (6.65E-06, 5.91E-05)	0.014
Hb	-7.995 (-12.389, -3.602)	<0.001
Body Temp	-0.874 (-4.399, 2.651)	0.627
Mixed Infection	-2.480 (-20.443, 15.484)	0.787

Regression coefficient (Coef), the 95% confidence interval (95% CI) and p-values based on robust regression model were generated. This analysis was used to asses association between gametocyte density, which is a continuous outcome and the outcomes of several variables as shown. Variables with and without body temp, which has been speculated to affect gametocyte density were separated in the analysis.

The duration was similar in those who had mixed infection versus those who did not (mean duration of 6.6 versus 6.3 days respectively, *P* = 0.8). None of the variables of interest had a statistically significant effect on the duration of gametocytaemia in the regression model.

## Discussion

This study presents longitudinal data on gametocytes carriage in natural infections in a cohort of children aged between 12 and 47 months old who were followed for one year. The data shows that treatment of asymptomatic parasitaemic subjects with AL results in a significant reduction in the proportion of subjects who become gametocytaemic for at least 12 weeks, despite AL having a relatively short half-life. The half-life of artemether is estimated to be 3.9 hrs and that of lumefantrine, the longer acting partner is estimated to be 32.7 hrs [36]. There was no effect seen in subjects who were not parasitaemic at enrolment. Studies comparing the risk of gametocyte carriage after treatment with AL versus dihydroartemisinin-piperaquine (DP) have had mixed results, with some studies showing no difference [37,38], some showing increased risk after treatment with AL [39,40] and DP [1,41-43]. Such differences might be accounted for by the detection method used in these studies and/or transmission intensity. Important also might be whether the study subjects were parasitaemic or not at the time of drug administration, which data here shows that this indeed matters. In a recent study, Sawa et al., showed that AL had a more pronounced effect on malaria transmission shortly after treatment compared to DP [44]. This was the first study to have directly determined gametocyte transmission to mosquitoes that fed on post-treatment blood samples. These authors speculated that AL may be the most appropriate first-line choice for reducing community-wide transmission of *P. falciparum* in setting of low endemicity. AL may be also a good candidate drug for mass screening and treatment of malaria as well as Focal screen and treat in the setting of high endemicity.

In line with other studies, the gametocyte prevalence dynamics throughout the study period followed closely that of the asexual parasite [11,27]. The maximum gametocytaemia duration has been estimated at 22.2 days [40] using microscopy and 107.7 days [41] using molecular methods, which was after non-ACT treatment of the subjects. The maximum gametocytaemia duration in this study was 44.5 days. It is plausible that parasite density was submicroscopic on days when it was recorded as zero and much longer gametocytes durations could have been estimated using molecular methods. This study showed no evidence of higher gametocyte density in the low malaria season as had been suggested before [1]. In areas of high malaria transmission intensity, gametocyte carriage is most prevalent in younger age groups, usually grouped as < 5 years old [11,27]. In this study, there were no variations of gametocyte prevalence between the age categories, further supporting clustering of < 5 years age group together in gametocyte prevalence analysis.

Having parasitaemia at enrolment and mixed infection had a statistically significant influence on the time to the occurrence of the first or only episode of gametocytaemia. These findings are in line with what previous studies have shown that mixed infection increases *P. falciparum* gametocyte production [29,45]. Unlike the previous studies that looked at co-infection with *P. malariae* only, the current study looked at co-infection with both *P. malariae* and/or *P. ovale*. Interestingly, being hyper parasitaemic or anaemic did not have a statistically significant influence on the time to the occurrence of the first or only event of gametocytaemia. Anaemia is considered to be a risk factor for gametocytaemia [2]. Although many factors have been speculated to be important in this relationship, cross-species immune responses seem to be the most plausible because hyper-parasitaemia or anaemia did not play a critical role. The study data presented here support a phenomenon where mixed infections trigger *P. falciparum* gametogenesis and hence increased transmission potential. Mixed infections therefore play an important role in prevalence and transmission of falciparum malaria.

This study had three main limitations: First, while the under-five age group was looked at exclusively, an important age category of this group - that of children aged below 1 year - was not looked at. The contribution of this age category in malaria transmission remains largely unknown. Second, there is lack of parasite molecular data that would have complemented the findings of this study. This is in light of the knowledge that sub-microscopic gametocytaemia plays a key role in malaria transmission, and that 3–10 fold increase in gametocyte carriage as determined by microscopy is seen when molecular detection methods are used [4,6-8,46]. Lastly, the original study was designed to address a different research question, and this may affect the statistical power of this study.

## Conclusion

This study provides key insights on the effect that treatment of asymptomatic carriers on malaria transmission. The treatment of asymptomatic parasitaemic subjects with AL results in a significant reduction in the proportion of subjects who become gametocytaemic for at least 12 weeks. There is no effect seen in subjects who are not parasitaemic at the time of drug administration.

## Competing interests

The authors declare that they have no competing interests.

## Authors’ contributions

BO was the study PI. BA, EK and BO conceived and executed the study (data analysis). BA and JM analysed the data. BA, JM, EK wrote the manuscript. BO reviewed the manuscript. All authors read and approved the final manuscript.
